# Supplementary material for: Chemosynthesis enables microbial communities to flourish in a marine cave ecosystem
Source: ISME J. 2025 Dec 23;20(1):wraf286. doi: 10.1093/ismejo/wraf286 (PMC12954392; doi:10.1093/ismejo/wraf286)
Supplement: wraf286_Supplemental_Files [file wraf286_supplemental_files.zip › Supp_Fig_5.pdf]

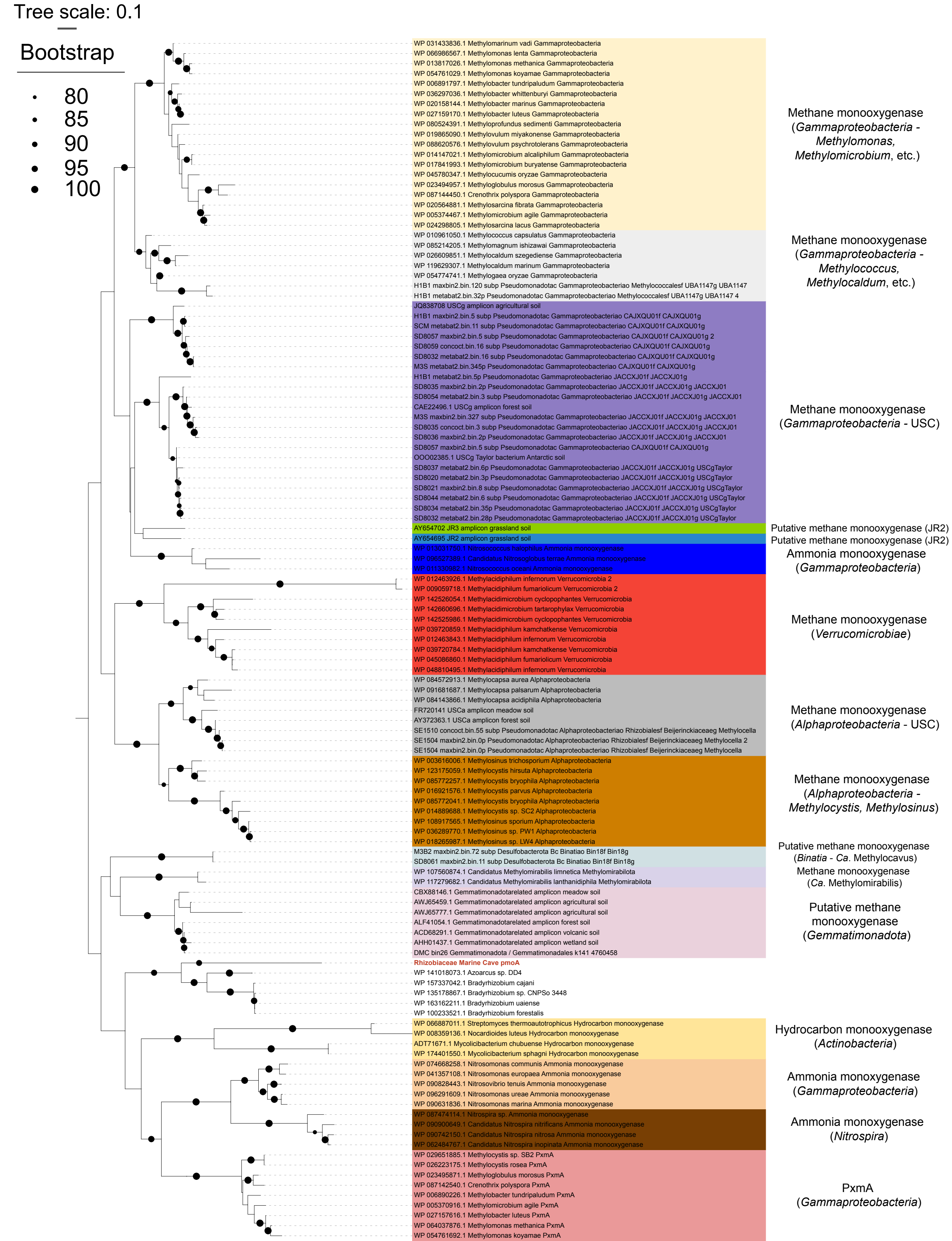
Tree scale: 0.1
Bootstrap
80
85
90
95
100
WP 031433836.1 Methylomarinum vadi Gammaproteobacteria
WP 066986567.1 Methylomonas lenta Gammaproteobacteria
WP 013817026.1 Methylomonas methanica Gammaproteobacteria
WP 054761029.1 Methylomonas koyamae Gammaproteobacteria
WP 006891797.1 Methylobacter tundripaludum Gammaproteobacteria
WP 036297036.1 Methylobacter whittenburyi Gammaproteobacteria
WP 020158144.1 Methylobacter marinus Gammaproteobacteria
WP 027159170.1 Methylobacter luteus Gammaproteobacteria
WP 080524391.1 Methyloprofundus sedimenti Gammaproteobacteria
WP 019865090.1 Methylovulum miyakonense Gammaproteobacteria
WP 088620576.1 Methylovulum psychrotolerans Gammaproteobacteria
WP 014147021.1 Methylomicrobium alcaliphilum Gammaproteobacteria
WP 017841993.1 Methylomicrobium buryatense Gammaproteobacteria
WP 045780347.1 Methylocucumis oryzae Gammaproteobacteria
WP 023494957.1 Methyloglobulus morosus Gammaproteobacteria
WP 087144450.1 Crenothrix polyspora Gammaproteobacteria
WP 020564881.1 Methylosarcina fibrata Gammaproteobacteria
WP 005374467.1 Methylomicrobium agile Gammaproteobacteria
WP 024298805.1 Methylosarcina lacus Gammaproteobacteria
Methane monooxygenase (Gammaproteobacteria - Methylomonas, Methylomicrobium, etc.)
WP 010961050.1 Methylococcus capsulatus Gammaproteobacteria
WP 085214205.1 Methylomagnum ishizawai Gammaproteobacteria
WP 026609851.1 Methylocaldum szegediense Gammaproteobacteria
WP 119629307.1 Methylocaldum marinum Gammaproteobacteria
WP 054774741.1 Methylogaea oryzae Gammaproteobacteria
H1B1 maxbin2.bin.120 subp Pseudomonadotac Gammaproteobacteriao Methylococcalesf UBA1147g UBA1147
H1B1 metabat2.bin.32p Pseudomonadotac Gammaproteobacteriao Methylococcalesf UBA1147g UBA1147 4
Methane monooxygenase (Gammaproteobacteria - Methylococcus, Methylocaldum, etc.)
JQ838708 USCg amplicon agricultural soil
H1B1 maxbin2.bin.5 subp Pseudomonadotac Gammaproteobacteriao CAJXQU01f CAJXQU01g
SCM metabat2.bin.11 subp Pseudomonadotac Gammaproteobacteriao CAJXQU01f CAJXQU01g
SD8057 maxbin2.bin.5 subp Pseudomonadotac Gammaproteobacteriao CAJXQU01f CAJXQU01g 2
SD8059 concoct.bin.16 subp Pseudomonadotac Gammaproteobacteriao CAJXQU01f CAJXQU01g
SD8032 metabat2.bin.16 subp Pseudomonadotac Gammaproteobacteriao CAJXQU01f CAJXQU01g
M3S metabat2.bin.345p Pseudomonadotac Gammaproteobacteriao CAJXQU01f CAJXQU01g
H1B1 metabat2.bin.5p Pseudomonadotac Gammaproteobacteriao JACCXJ01f JACCXJ01g
SD8035 maxbin2.bin.2p Pseudomonadotac Gammaproteobacteriao JACCXJ01f JACCXJ01g JACCXJ01
SD8054 metabat2.bin.3 subp Pseudomonadotac Gammaproteobacteriao JACCXJ01f JACCXJ01g JACCXJ01
CAE22496.1 USCg amplicon forest soil
M3S maxbin2.bin.327 subp Pseudomonadotac Gammaproteobacteriao JACCXJ01f JACCXJ01g JACCXJ01
SD8035 concoct.bin.3 subp Pseudomonadotac Gammaproteobacteriao JACCXJ01f JACCXJ01g JACCXJ01
SD8036 maxbin2.bin.2p Pseudomonadotac Gammaproteobacteriao JACCXJ01f JACCXJ01g JACCXJ01
SD8057 maxbin2.bin.5 subp Pseudomonadotac Gammaproteobacteriao CAJXQU01f CAJXQU01g
OOO02385.1 USCg Taylor bacterium Antarctic soil
SD8037 metabat2.bin.6p Pseudomonadotac Gammaproteobacteriao JACCXJ01f JACCXJ01g USCgTaylor
SD8020 metabat2.bin.3p Pseudomonadotac Gammaproteobacteriao JACCXJ01f JACCXJ01g USCgTaylor
SD8021 maxbin2.bin.8 subp Pseudomonadotac Gammaproteobacteriao JACCXJ01f JACCXJ01g USCgTaylor
SD8044 metabat2.bin.6 subp Pseudomonadotac Gammaproteobacteriao JACCXJ01f JACCXJ01g USCgTaylor
SD8034 metabat2.bin.35p Pseudomonadotac Gammaproteobacteriao JACCXJ01f JACCXJ01g USCgTaylor
SD8032 metabat2.bin.28p Pseudomonadotac Gammaproteobacteriao JACCXJ01f JACCXJ01g USCgTaylor
Methane monooxygenase (Gammaproteobacteria - USC)
AY654702 JR3 amplicon grassland soil
Putative methane monooxygenase (JR2)
AY654695 JR2 amplicon grassland soil
Putative methane monooxygenase (JR2)
WP 013031750.1 Nitrosococcus halophilus Ammonia monooxygenase
WP 096527389.1 Candidatus Nitrosoglobus terrae Ammonia monooxygenase
WP 011330982.1 Nitrosococcus oceani Ammonia monooxygenase
Ammonia monooxygenase (Gammaproteobacteria)
WP 012463926.1 Methylacidiphilum infernorum Verrucomicrobia 2
WP 009059718.1 Methylacidiphilum fumariolicum Verrucomicrobia 2
WP 142526054.1 Methylacidimicrobium cyclopophantes Verrucomicrobia
WP 142660696.1 Methylacidimicrobium tartarophylax Verrucomicrobia
WP 142525986.1 Methylacidimicrobium cyclopophantes Verrucomicrobia
WP 039720859.1 Methylacidiphilum kamchatkense Verrucomicrobia
WP 012463843.1 Methylacidiphilum infernorum Verrucomicrobia
WP 039720784.1 Methylacidiphilum kamchatkense Verrucomicrobia
WP 045086860.1 Methylacidiphilum fumariolicum Verrucomicrobia
WP 048810495.1 Methylacidiphilum infernorum Verrucomicrobia
Methane monooxygenase (Verrucomicrobiae)
WP 084572913.1 Methylocapsa aurea Alphaproteobacteria
WP 091681687.1 Methylocapsa palsarum Alphaproteobacteria
WP 084143866.1 Methylocapsa acidiphila Alphaproteobacteria
FR720141 USCa amplicon meadow soil
AY372363.1 USCa amplicon forest soil
SE1510 concoct.bin.55 subp Pseudomonadotac Alphaproteobacteriao Rhizobialesf Beijerinckiaceaeg Methylocella
SE1504 maxbin2.bin.0p Pseudomonadotac Alphaproteobacteriao Rhizobialesf Beijerinckiaceaeg Methylocella 2
SE1504 maxbin2.bin.0p Pseudomonadotac Alphaproteobacteriao Rhizobialesf Beijerinckiaceaeg Methylocella
Methane monooxygenase (Alphaproteobacteria - USC)
WP 003616006.1 Methylosinus trichosporium Alphaproteobacteria
WP 123175059.1 Methylocystis hirsuta Alphaproteobacteria
WP 085772257.1 Methylocystis bryophila Alphaproteobacteria
WP 016921576.1 Methylocystis parvus Alphaproteobacteria
WP 085772041.1 Methylocystis bryophila Alphaproteobacteria
WP 014889688.1 Methylocystis sp. SC2 Alphaproteobacteria
WP 108917565.1 Methylosinus sporium Alphaproteobacteria
WP 036289770.1 Methylosinus sp. PW1 Alphaproteobacteria
WP 018265987.1 Methylosinus sp. LW4 Alphaproteobacteria
Methane monooxygenase (Alphaproteobacteria - Methylocystis, Methylosinus)
M3B2 maxbin2.bin.72 subp Desulfobacterota Bc Binatiao Bin18f Bin18g
SD8061 maxbin2.bin.11 subp Desulfobacterota Bc Binatiao Bin18f Bin18g
Putative methane monooxygenase (Binatia - Ca. Methylocavus)
WP 107560874.1 Candidatus Methylomirabilis limnetica Methylomirabilota
WP 117279682.1 Candidatus Methylomirabilis lanthanidiphila Methylomirabilota
Methane monooxygenase (Ca. Methylomirabilis)
CBX88146.1 Gemmatimonadotarelated amplicon meadow soil
AWJ65459.1 Gemmatimonadotarelated amplicon agricultural soil
AWJ65777.1 Gemmatimonadotarelated amplicon agricultural soil
ALF41054.1 Gemmatimonadotarelated amplicon forest soil
ACD68291.1 Gemmatimonadotarelated amplicon volcanic soil
AHH01437.1 Gemmatimonadotarelated amplicon wetland soil
DMC bin26 Gemmatimonadota / Gemmatimonadales k141 4760458
Putative methane monooxygenase (Gemmatimonadota)
Rhizobiaceae Marine Cave pmoA
WP 141018073.1 Azoarcus sp. DD4
WP 157337042.1 Bradyrhizobium cajani
WP 135178867.1 Bradyrhizobium sp. CNPSo 3448
WP 163162211.1 Bradyrhizobium uaiense
WP 100233521.1 Bradyrhizobium forestalis
WP 066887011.1 Streptomyces thermoautotrophicus Hydrocarbon monooxygenase
WP 008359136.1 Nocardioides luteus Hydrocarbon monooxygenase
ADT71671.1 Mycolicibacterium chubuense Hydrocarbon monooxygenase
WP 174401550.1 Mycolicibacterium sphagni Hydrocarbon monooxygenase
Hydrocarbon monooxygenase (Actinobacteria)
WP 074668258.1 Nitrosomonas communis Ammonia monooxygenase
WP 041357108.1 Nitrosomonas europaea Ammonia monooxygenase
WP 090828443.1 Nitrosovibrio tenuis Ammonia monooxygenase
WP 096291609.1 Nitrosomonas ureae Ammonia monooxygenase
WP 090631836.1 Nitrosomonas marina Ammonia monooxygenase
Ammonia monoxygenase (Gammaproteobacteria)
WP 087474114.1 Nitrospira sp. Ammonia monooxygenase
WP 090900649.1 Candidatus Nitrospira nitrificans Ammonia monooxygenase
WP 090742150.1 Candidatus Nitrospira nitrosa Ammonia monooxygenase
WP 062484767.1 Candidatus Nitrospira inopinata Ammonia monooxygenase
Ammonia monooxygenase (Nitrospira)
WP 029651885.1 Methylocystis sp. SB2 PxmA
WP 026223175.1 Methylocystis rosea PxmA
WP 023495871.1 Methyloglobulus morosus PxmA
WP 087142540.1 Crenothrix polyspora PxmA
WP 006890226.1 Methylobacter tundripaludum PxmA
WP 005370916.1 Methylomicrobium agile PxmA
WP 027157616.1 Methylobacter luteus PxmA
WP 064037876.1 Methylomonas methanica PxmA
WP 054761692.1 Methylomonas koyamae PxmA
PxmA (Gammaproteobacteria)
