# Supplementary figures and images for: Chemosynthesis enables microbial communities to flourish in a marine cave ecosystem
Source: ISME J. 2025 Dec 23;20(1):wraf286. doi: 10.1093/ismejo/wraf286 (PMC12954392; doi:10.1093/ismejo/wraf286)

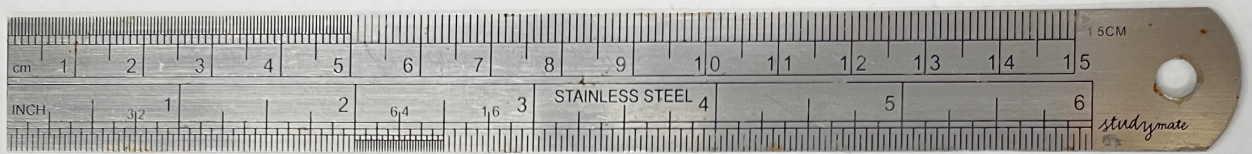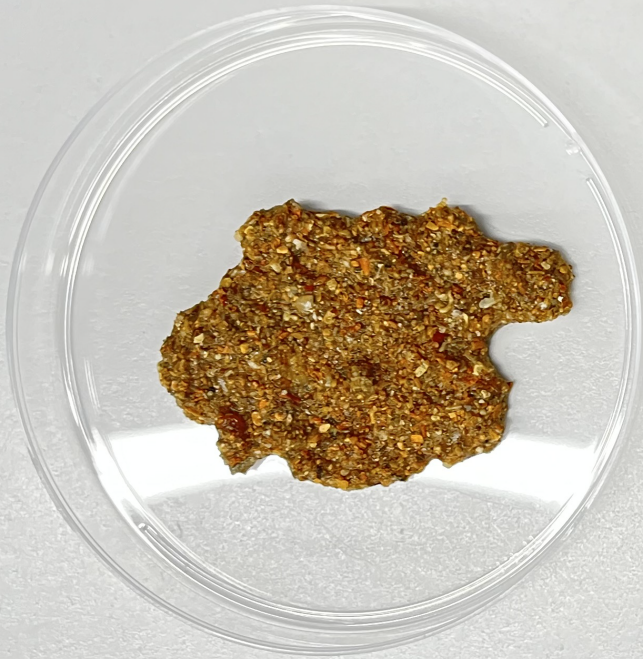

ENTRANCE

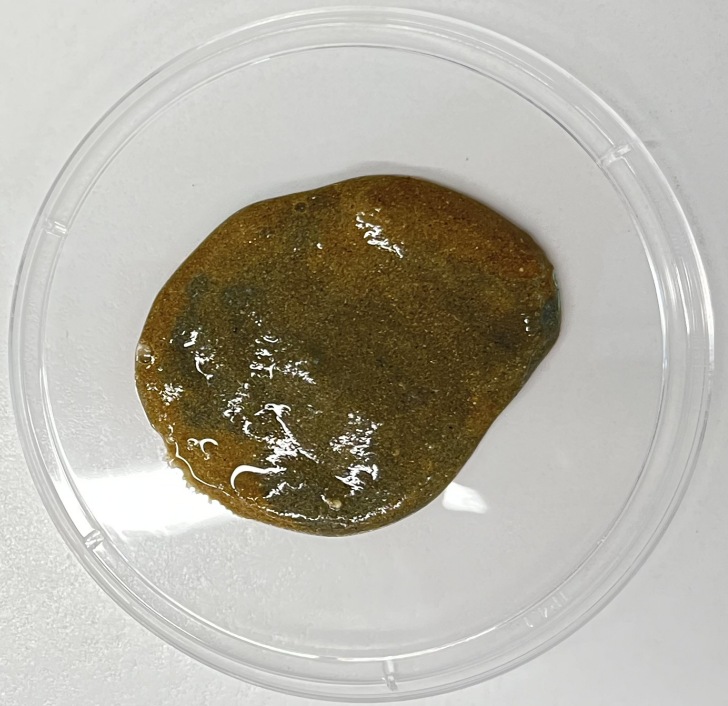

INNER

Supplement: wraf286_Supplemental_Files [file wraf286_supplemental_files.zip › Supp_Fig_1_wraf286.pdf]

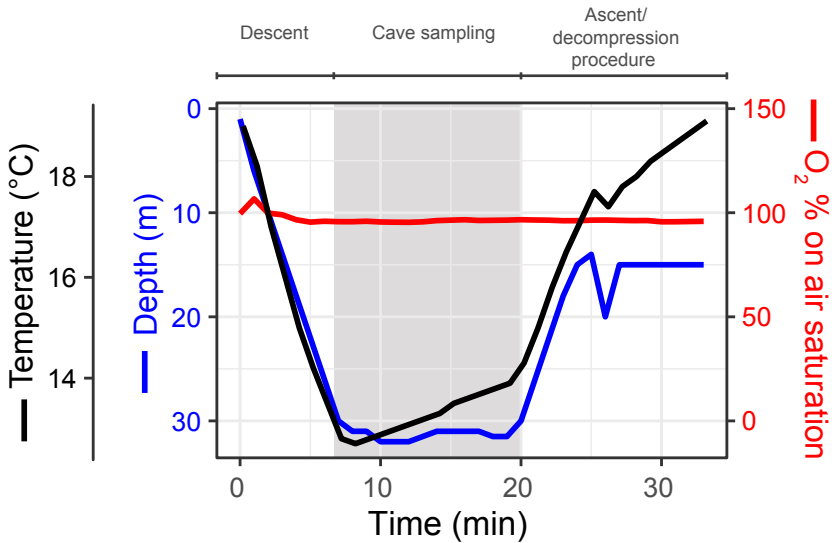

Supplement: wraf286_Supplemental_Files [file wraf286_supplemental_files.zip › Supp_Fig_2_wraf286.pdf]

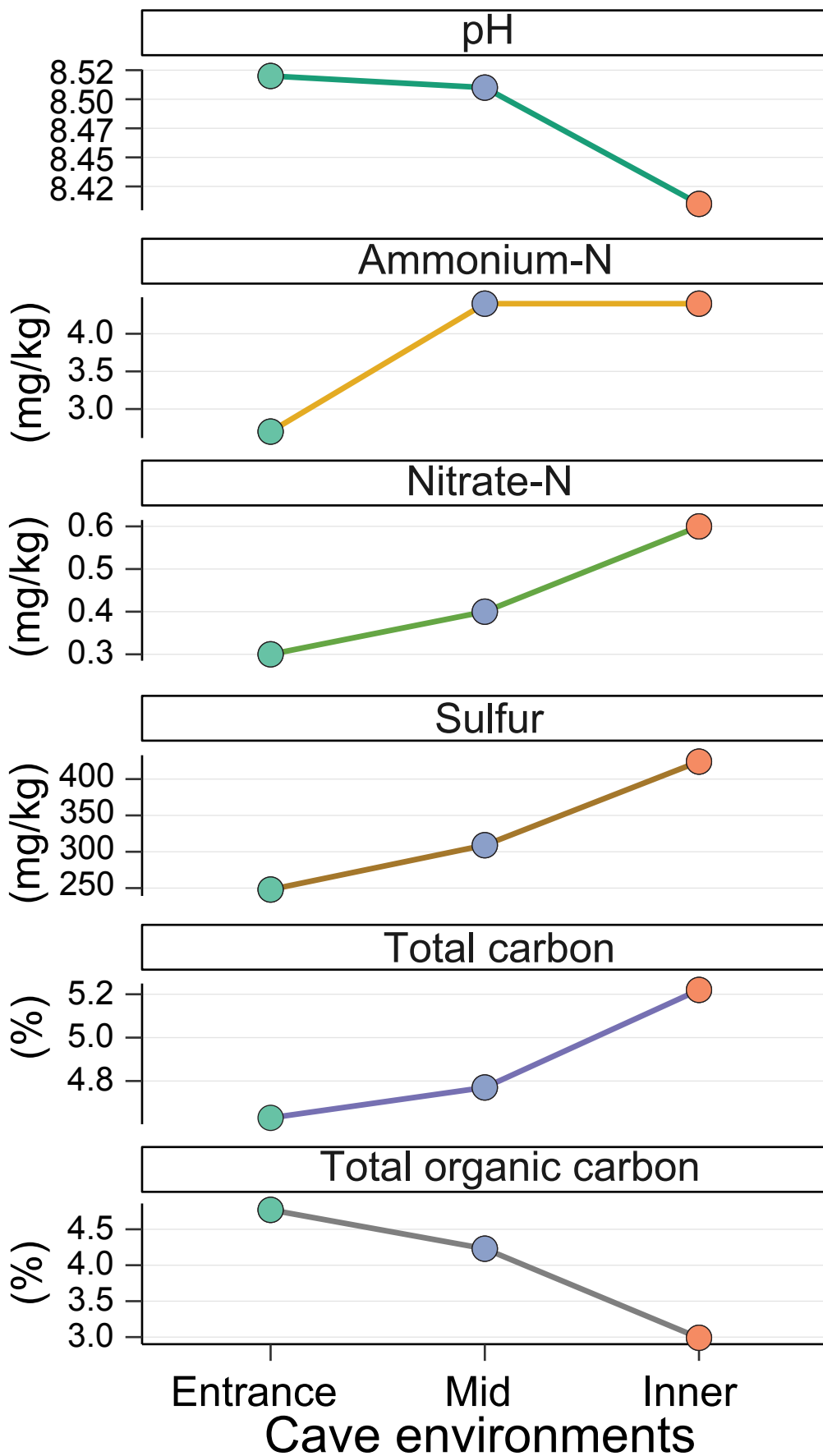

Supplement: wraf286_Supplemental_Files [file wraf286_supplemental_files.zip › Supp_Fig_3_wraf286.pdf]

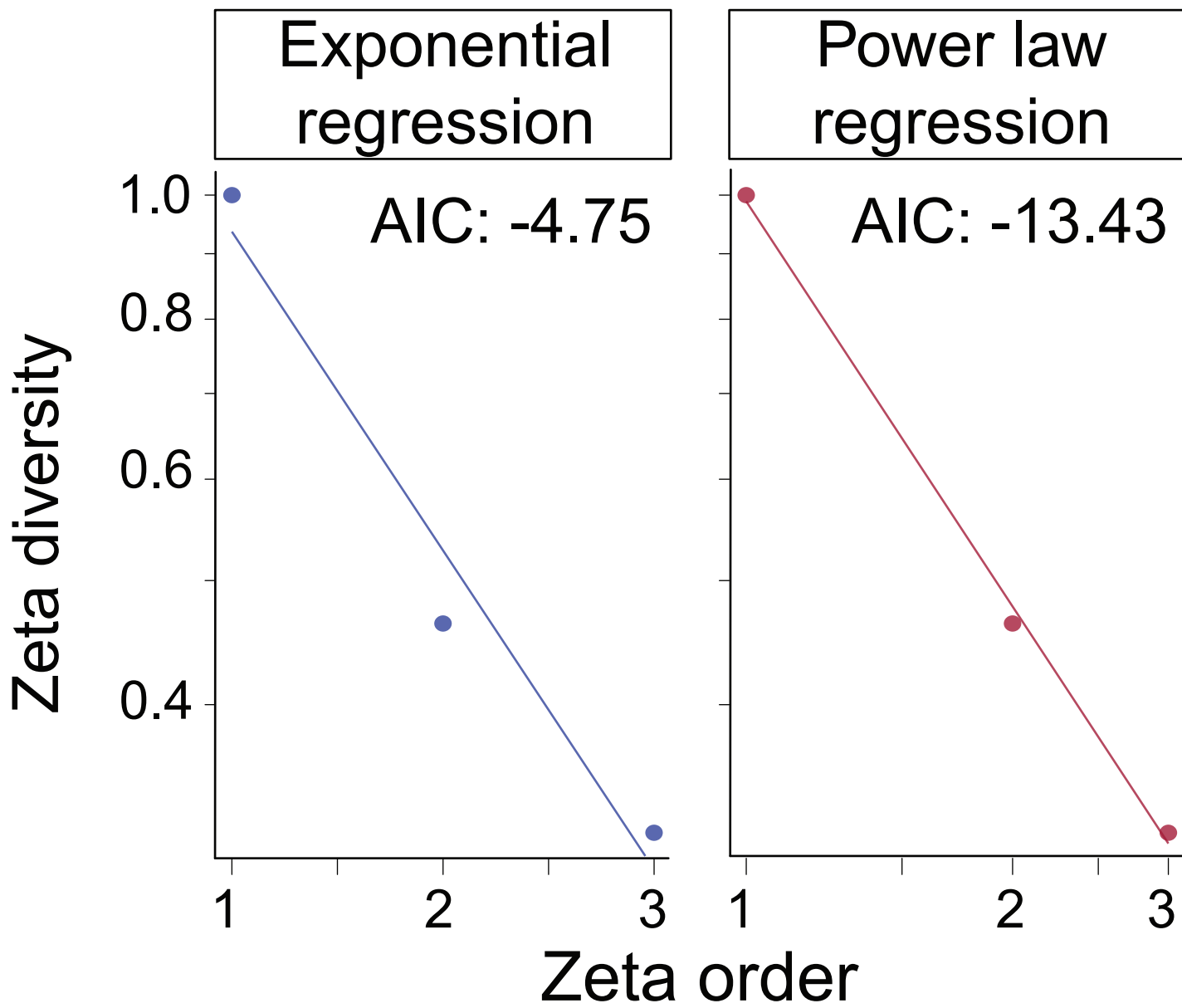

Supplement: wraf286_Supplemental_Files [file wraf286_supplemental_files.zip › Supp_Fig_4_wraf286.pdf]
